# Immediate neck hypoalgesic effects of craniocervical flexion exercises and cervical retraction exercises among individuals with non-acute neck pain and a directional preference for retraction or extension: preliminary pretest-posttest randomized experimental design

**DOI:** 10.1080/10669817.2023.2201918

**Published:** 2023-04-13

**Authors:** Hiroshi Takasaki, Chisato Yamasaki

**Affiliations:** Department of Physical Therapy, Saitama Prefectural University, Koshigaya, Saitama, Japan

**Keywords:** Analgesics, exercise, neck, pain threshold

## Abstract

**Background:**

Selective deep neck flexor muscle activation through craniocervical flexion exercises has been considered to be different from cervical retraction exercises.

**Objective:**

To compare the immediate analgesic effect of craniocervical flexion versus cervical retraction exercises in individuals with nonacute, directional preference (DP) for cervical retraction or extension

**Methods:**

A two-arm, assessor-blinded, pretest-posttest randomized experiment was conducted. Participants were randomly assigned to either craniocervical flexion or cervical retraction exercises and those who were confirmed at the post-intervention examination to have a DP for cervical retraction or extension were analyzed. The primary outcome measure was pressure pain thresholds at the C2 and C5-C6 levels.

**Results:**

A total of 10 (mean age = 20.6 years) and nine participants (mean age = 19.4 years) undertook craniocervical flexion and retraction exercises, respectively. One-way analysis of variance demonstrated no statistically significant (*p* > 0.05) interaction effect regardless of the neck level. In the pre-post change percentages, retraction exercises provided greater analgesic effects compared to craniocervical flexion exercises at the C2 (Hedges’ g = 0.679) and C5-C6 levels (g = 0.637).

**Conclusion:**

This study showed a comparable or greater immediate neck analgesic effect from cervical retraction exercises compared to craniocervical flexion exercises in individuals with a DP for cervical retraction or extension.

## Introduction

Therapeutic exercise is consistently recommended for neck pain management in various clinical practice guidelines [1]. Craniocervical flexion exercises aimed at selective deep neck flexor (DNFs) muscle activation have been determined to be an effective exercise regimen in reducing neck pain and disability [2–5], even though opinions differ as to which exercises are most effective.

Craniocervical flexion exercise has an immediate local analgesic effect on the neck region [6–8]. Additionally, the immediate analgesic effect is greater with craniocervical flexion exercise than with passive cervical spine joint mobilization with minimal craniocervical flexion exercise [8] and isometric co-contractions of the superficial and DNFs [7]. Based on these findings, selective DNF activation has been considered unique and distinct from other cervical exercises, including cervical retraction exercises that activates the deep and superficial neck flexor muscles [9]. On the other hand, a study using pressure pain threshold (PPT) reported that immediate local neck analgesia can be provided by just imagining neck exercises composed of craniocervical flexion and cervical retraction exercises [10]. This suggests that the pain-suppressing function of the cognitive process of motor imagery in the brain may be involved in the mechanism of the pain-relieving effect of craniocervical flexion exercise. Therefore, it is unclear if the analgesic effect of selective DNF activation is superior to cervical retraction exercise in individuals with neck pain. It has been reported that increased DNF activation is associated with greater pain reduction [11]. Cervical retraction accompanies the greatest upper cervical spine flexion [12] and is expected to involve greater DNF activation than craniocervical flexion exercise with selective but low load DNF activation [9]. Therefore, cervical retraction exercise may have a more immediate local analgesic effect on the neck than craniocervical flexion exercise; however, this hypothesis has not been investigated to date.

The McKenzie Method of Mechanical Diagnosis and Therapy (MDT) is a musculoskeletal management system that frequently utilizes cervical retraction exercise for the management of neck pain. In MDT, classification is guided by the patient’s history and symptom responses to mechanical loading strategies (e.g. repeated movements or sustained postures). Derangement syndrome is a primary MDT classification and has a directional preference (DP) that is a specific mechanical loading strategy direction resulting in instantaneous and sustained improvement of symptoms, including centralization. Cervical retraction involves moderate segmental extension of the lower cervical spine [12] and is commonly used as an initial mechanical loading strategy for those with the most common DP for cervical extension [13]. The MDT classification of ‘Other’ is utilized when there is no DP and acute inflammatory pain causes aggravation with movement.

The Patient-Centered Outcomes Research Institute stated that one of the research priorities in neck pain includes exercise effectiveness along the DP [14], which has not been determined in the current literature due to a lack of high-quality research using a population with a DP [15]. Thus, future studies should examine a cohort with a DP. Therefore, this study aimed to preliminarily compare the immediate local analgesic effect on the neck region between exercise regimens using craniocervical flexion and cervical retraction in individuals with non-acute neck symptoms with a DP for cervical retraction or extension. We hypothesized that cervical retraction exercises would result in a similar or greater immediate local analgesic effect on the neck region than the craniocervical flexion exercises in this population.

## Methods

### Design

This is a two-arm, assessor-blinded, pretest-posttest randomized experimental study. Ethical approval was obtained from an institutional research ethics committee (Saitama Prefectural University; #21006). This study was registered at the University Hospital Medical Information Network (R000050748) and participants provided written informed consent before data collection.

### Participants

Participants were recruited via university e-mail from June 2021 to November 2021. The inclusion criteria were university students aged>17 years with a Neck Disability Index (NDI) [16] of≥16% [17]. The exclusion criteria were as follows: 1) neck symptom onset<1 week, an acute phase; 2) diagnosed specific pathologies of neck symptoms (e.g. cervical spondylosis); 3) painful symptoms other than neck pain with and without neck-shoulder stiffness; 4) symptoms indicating serious pathologies, such as dizziness, nystagmus, and dysarthria; 5) trauma history (i.e. neck pain onset after a traumatic event, such as a traffic accident), surgery, or spine or upper limb fracture; 6) currently undertaking any interventions other than massage and cold/hot patch for the neck symptom; and 7) those who did not have a DP for cervical retraction or extension at a post-intervention examination. Acute pain was excluded due to concerns that the pain mechanism might involve inflammation that could be exacerbated by mechanical neck loading. The operational definition of the derangement syndrome with a DP of cervical retraction or extension was the symptom with instantaneous and sustained improvement, including centralization in response to the mechanical loading strategies of cervical retraction or extension, including repetitive active movements with and without a therapist’s overpressure and static loading (e.g. holding a retracted neck position for a certain duration). Because symptoms may change during the process of diagnosing the derangement syndrome, confirmation of the derangement syndrome cannot be included in the inclusion criteria before data collection and the diagnosis had to be made after data collection completion.

Regarding sample size, a previous study with a sample size of 18 [8] showed a statistically significant main effect for the time (*F* = 4.3, *P* = 0.04) of the immediate improvement in PPT over C5 with passive joint mobilization and craniocervical flexion exercise. Therefore, this study decided on a final sample size with 18 participants who had the derangement syndrome with a DP of cervical retraction or extension. In the general population, the percentage of patients with the derangement syndrome has been reported to be 82% [18]. Acute and/or traumatic neck pain was excluded in the current study. Thus, the percentage of the participants who had the derangement syndrome with a DP for cervical retraction or extension was expected to be 90% in the current study. As a result, 20 participants were included in the hopes of capturing 18 participants who had the derangement syndrome with a DP for cervical retraction or extension.

### Therapist

Interventions were provided by the first author (HT) who has been a licensed physical therapist for 18 years. He has published articles using the craniocervical flexion test [19,20]. He is also a credentialed MDT therapist, who has demonstrated interexaminer reliability in the MDT classification for spinal problems [21], and has completed MDT diploma clinical training.

### Intervention

A session of exercise with craniocervical flexion or cervical retraction was performed individually. Each group’s exercise time was 3 min per set based on the protocol in a previous study [8] for a total of three sets of 9 min each, with a 3-min break between each set.

In the craniocervical flexion exercise group, participants were in the supine, knee-flexed position with the cervical spine in neutral position. Repeated craniocervical flexion movements with the use of a pressure feedback device (Stabilizer, Chattanooga, TN, USA) were performed. First, based on an established craniocervical flexion test procedure [22], the exercise level that each participant could perform correctly was determined (22, 24, 26, 28, and 30 mmHg). Subsequently, participants repeated craniocervical flexion at a rhythm of one repetition per 2 s for 3 min as per the protocol in a previous study [8]. During the exercise, verbal instructions, as well as visual feedback from pressure biofeedback, were provided to help participants perform the correct craniocervical flexion exercise. During each 3-min break, participants rested in the supine position without any movement. The craniocervical flexion test is said to be a reliable and valid procedure for assessing DNF performance and correct craniocervical flexion exercises activate DNF with minimal contraction of the superficial neck muscles [23–25].

In the cervical retraction exercise group, participants sat in an upright posture. Participants repeated cervical retraction at a rhythm of one repetition every 2 s for 3 min with self-over pressure at end-range. During the exercise, verbal instructions were given to participants to perform the correct cervical retraction exercise, however, if they still could not do it correctly, the therapist guided the movement as minimally as appropriate. During each 3-min break, participants rested in a sitting position without any movements.

### Outcome measures

PPT was the primary outcome measure using a computerized pressure algometer (Algomed, Medoc Ltd., Ramat Yishay, Israel) according to the protocol used in a previous study [8]. A 1 cm^2^ surface contact area and pressure were applied perpendicularly to the skin with a 45° angle between the frontal and sagittal planes at a rate of 1 kg/cm^2^/s using a visual pressure guide. PPT was assessed at a posterolateral location between the occiput’s lower border and the horizontal level of the spinous process of C2 and over the C5-C6 zygapophyseal joint by a blinded assessor (Assessor 1, CY). The measured side was the most painful laterality identified by each participant and the right side was tested when pain intensity is equal between both sides based on the previous study’s protocol [8]. PPT was assessed before randomization and immediately after the intervention. PPT was measured four times consecutively at each location with 30 s of rest between measurements after an explanation of the measurement and demonstration on the forearm. The first PPT measurement was discarded and the mean of the subsequent three PPT measurements were used for further analysis.

The following secondary outcome measures were assessed at baseline before randomization: 1) demographic information (age, gender, and dominant hand), 2) symptom duration, 3) pain intensity based on the 4-item Pain Intensity Measure (P4), 4) quality of life according to the EuroQol-5 Dimension (EQ-5D), and 5) disability according to NDI (%). Symptom duration (months) was defined as the time since the last symptom-free month [26]. P4 is a reliable 11-point numerical rating scale (NRS) for pain intensity, with a higher sum score indicating greater pain intensity (0–40) [27]. The EQ-5D [28] includes five items (mobility, self-care, usual activities, pain or discomfort, and anxiety or depression). It has been considered a reliable and valid patient-reported outcome measure (PROM) of quality of life. A higher score indicates better quality of life (0–1) [29]. NDI is a reliable and valid PROM of disability due to neck pain [30], with a higher percentage score indicating greater disability.

The following outcomes of 1) neck range of motion (ROM), 2) current pain intensity, and 3) symptom location were assessed before randomization and immediately after the intervention. Neck ROM was assessed using the Cervical Range of Motion (CROM) instrument by a blinded assessor (Assessor 2, SN) using standardized movement instructions. Neck ROM was measured thrice each in flexion, extension, left and right lateral bending, and left and right rotation. The sum of the means was calculated [31]. Adequate inter-session and inter-examiner reliabilities of the neck ROM using the CROM were reported in previous studies [32–35]. Current pain intensity at the most painful location in the resting sitting position was assessed using a 0–10 NRS. Symptom location in the resting sitting position where a greater sum indicated more widespread symptoms (0–14) was assessed using an upper body chart with 14 parts [36–38]. The sum number of the body parts was calculated.

### Randomization

Cards describing the intervention for 10 participants in each of the two groups were sealed in the same nontransparent envelope and kept by a research assistant for randomization throughout the study. After the baseline assessment, the participants withdrew one envelope from the lottery box where all the envelopes were placed. In the presence of the randomization assistant and intervention therapist, participants read the card for the intervention and the written intervention was implemented. The intervention allocation was concealed from all assessors of objective outcome measures (i.e. PPT and cervical ROM) throughout the study to maintain assessor blinding.

All participants took MDT examinations with physiotherapist (HT) after data collection of post-intervention outcomes using mechanical loading strategies to the cervical spine to identify an MDT classification. Data of outcomes were blinded to the physiotherapist (HT) until an MDT classification was identified.

### Data analysis

Descriptive analysis was used to summarize the data. Interactions between groups × time and pre-post and group effects were determined using one-way repeated measures analysis of variance (ANOVA) for the following outcomes: PPT, neck ROM, current pain intensity, and symptom location. Further, pre-post change percentage in these outcomes was calculated and the pre-post effect was compared between the two groups using Hedges’ *g*. Demographic group differences were examined using independent samples t-test or Fisher’s exact test. IBM SPSS Statistics for Windows, Version 28.0 (Armonk, NY: IBM Corp) was used to conduct statistical analysis with a 5% significance level. The effect size of the Hedges’ *g* was interpreted as small at 0.2, moderate at 0.5, and large at 0.8.

## Results

**Figure 1.**
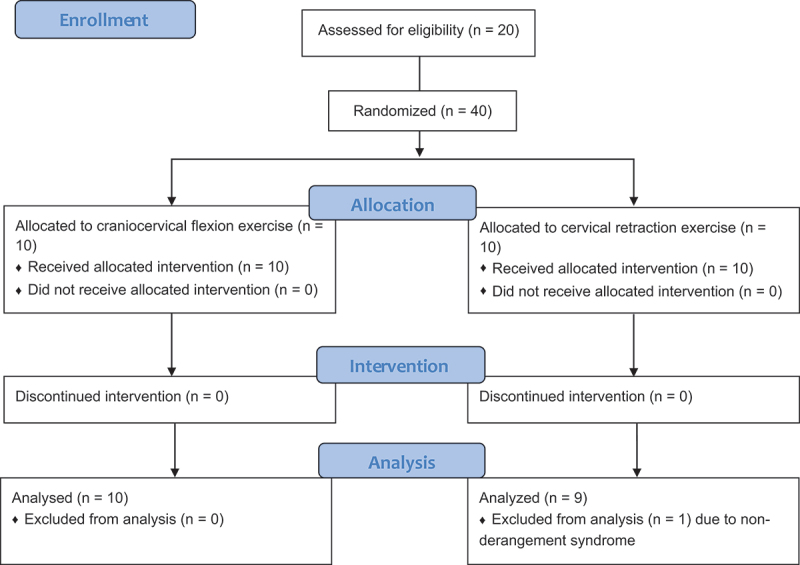
The flow of the participant.

**Figure 2.**
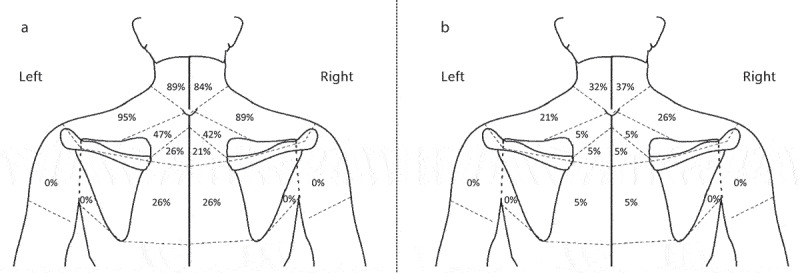
Symptom distributions.

Out of the 20 participants, a participant in the cervical retraction group was classified into the ‘Other’ MDT classification and was thus excluded from data analysis (Figure 1). There were neither adverse events nor dropouts noted in this study. Demographics are summarized in Table 1 and Figure 2. In the craniocervical flexion group, the craniocervical flexion levels were 22 mmHg in six participants, 24 mmHg in two participants, and 26 mmHg in two participants. Regarding the MDT classification identified at the postintervention examination, centralization with a DP for cervical retraction or extension was confirmed in all participants in the craniocervical flexion exercise group. Among the 19 participants who had the derangement syndrome with a DP for cervical retraction or extension in the cervical retraction exercise group, seven participants had centralization and two without centralization.

A: before randomization, B: immediately after the interventions

**Table 1.** Demographics of participants.

Variables	Craniocervical flexion exercise group (*n* = 10)	Cervical retraction exercise group (*n* = 9)	Group differences (*p*-value)
Age (year)*	20.6 (3.1)	19.4 (1.7)	0.338
Gender†	2 men, 8 women	9 women	0.474
Dominant hand side†	2 left, 8 right	9 right	0.474
Symptom duration (month)*	20.1 (23.9)	49.7 (51.0)	0.140
4-item Pain Intensity Measure (0–40)*	14.9 (6.5)	21.0 (3.4)	0.022
EuroQol 5 Dimension (0–1)*	0.71 (0.06)	0.73 (0.03)	0.227
Neck Disability Index (%)*	27.0 (7.5)	29.9 (14.1)	0.577

Values are presented with a mean (standard deviations) unless specified.

*Independent samples t-test.

†Fisher’s exact test.

Statistically significant one-way repeated measures ANOVAs demonstrated pre-post effects in the PPT, neck ROM, current pain intensity, and symptom location and a statistically significant interaction effect in neck ROM (Table 2). Regarding effect sizes of group differences in the percentage of pre-post changes, greater hypoalgesia and neck ROM improvement with more than moderate effect sizes were detected in the cervical retraction exercise group (Table 3).

**Table 2.** The pre-post difference in primary and secondary outcomes.

Variables	Craniocervical flexion exercise group (*n* = 10)		Cervical retraction exercise group (*n* = 9)		Interaction effect (*p*-value)
	Before randomization	Immediately after an intervention	Before randomization	Immediately after an intervention	
PPT at the C2 level (kPa)	162.3 (66.3)	172.7 (71.9)	149.2 (60.5)	196.1 (89.5)	0.194*
PPT at the C5/6 level (kPa)	143.0 (54.3)	178.7 (61.9)	148.5 (39.2)	214.8 (43.3)	0.146†
Summed neck range of motion (°)	311.0 (33.9)	320.3 (45.2)	295.5 (54.6)	351.3 (51.9)	0.014‡
Current pain intensity (0–10)	2.9 (2.2)	1.4 (1.6)	3.6 (2.4)	1.8 (1.3)	0.558§
Symptom location (0–14)	4.7 (1.2)	1.4 (1.3)	6.3 (2.7)	1.6 (1.9)	0.146‖

Values are presented with mean (standard deviations) unless specified.

Abbreviations: PPT, pressure pain threshold.

*pre-post effect: *p* = 0.048; group effect: *p* = 0.868.

†pre-post effect: *p* < 0.001; group effect: *p* = 0.339.

‡pre-post effect: *p* = 0.001; group effect: *p* = 0.700.

§pre-post effect: *p* = 0.0001; group effect: *p* = 0.623.

‖pre-post effect: *p* < 0.001; group effect: *p* = 0.218.

**Table 3.** The effect size of group differences in % pre-post favorable changes.

Variables	Craniocervical flexion exercise group (*n* = 10)	Cervical retraction exercise group (*n* = 9)	Hedges *g* [95% CIs]
% pre-post increase of PPT at the C2 level	9.0 (20.0)	44.0 (68.7)	0.679 [−0.220 to 1.559]
% pre-post increase of PPT at the C5/6 level	27.5 (28.4)	54.5 (50.7)	0.637 [−0.257 to 1.515]
% pre-post increase of summed neck range of motion	3.0 (20.7)	9.2 (17.0)	1.259 [0.287 to 2.201]
% pre-post decrease of current pain intensity	48.7 (37.8)	45.6 (37.7)	0.079 [−0.783 to 0.938]
% pre-post decrease of symptom location	71.7 (26.1)	76.5 (23.8)	0.184 [−0.681 to 1.043]

Values are presented with a mean (standard deviations).

Abbreviations: PPT, pressure pain threshold; CIs, confidence intervals.

*Greater favorable effect to the cervical retraction exercise group than the craniocervical flexion exercise group are presented with positive values.

## Discussion

This preliminary study compared the immediate local analgesic effect on the neck region between the craniocervical flexion exercise and the cervical retraction exercise in those who had the non-acute derangement syndrome with a DP of cervical retraction or extension. In PPT, pre-intervention PPTs were similar to some previous studies using similar neck measurement locations in people with chronic neck pain [8,39] and the percent pre-post increase of PPTs in the craniocervical flexion exercise also seems to be similar to a previous study (C2 level = 19.5%, C5-C6 level = 10.2%) [8]. These support external validity of the findings in the current study. Importantly, there was a statistically significant pre-post effect indicating an analgesic effect at each neck level, although there was no statistically significant interaction effect at each neck level. Additionally, when the pre-post increase percentage of PPT was compared between the two exercise groups, a moderate effect size in favor of cervical retraction exercise was detected at each neck level. These findings indicate comparable or greater analgesic effects with cervical retraction than with craniocervical flexion exercises in this study’s population, supporting our hypothesis. Motor imagery of craniocervical flexion and cervical retraction exercises may impart an analgesic effect [10]. This point should be investigated further in future studies.

Regarding pain intensity, as well as pain location, statistically significant pre-post improvements were detected in both exercise regimens. In particular, the pain intensity decreased by half for both exercise groups after 9 min of exercise. PPT as well as pre- and post-intervention pain intensities in the 11-point numerical pain rating scale seems to be comparable to the study by Llch et al. [8] (pre-intervention pain intensity = 3.3 ± 1.8, postintervention pain intensity = 1.2 ± 1.3). The researchers used only one 3-min set craniocervical flexion exercises whereas this study used a total of 9-min craniocervical flexion exercise. Although further verification is needed by considering both immediate and long-term effects, from a dose-response perspective, craniocervical flexion exercises may, to some extent, reach a plateau for the activation of immediate pain-suppressing function for the neck region at about 3 min.

Regarding neck ROM, a statistically significant interaction effect was detected between the two exercise regimens with cervical retraction exercise providing a greater percentage of improvement with a large effect size (*g* = 1.259) compared with craniocervical flexion exercise. These findings may support the validity of using retraction exercises and end-range mechanical loading strategies to improve ROM in individuals who have the derangement syndrome with a DP of cervical retraction or extension. Similar findings have been reported in a randomized controlled trial in individuals with low back pain [40].

In the current study, one participant with an ‘Other’ MDT classification was identified and excluded from the analysis. A total of 19/20 participants (95%) had the derangement syndrome with a DP for cervical retraction or extension, in which an 82% derangement syndrome rate was reported in a cohort study [18]. Although classifications other than the derangement syndrome are in the minority, it would be required to examine the effects of neck exercise regimens in those individuals using a larger cohort in the future.

### Limitations

This study has several limitations. The first limitation is the use of convenience sampling and a small cohort, which biased the participants toward young women. Thus, the findings in this study should be interpreted carefully as preliminary evidence. Also considering the possibility of type II errors, validation with a larger cohort would be needed to draw definitive conclusions. However, we used an NDI cutoff of 16%, a disability level similar to those who need medical care in Japan [17]. Therefore, it is believed that, to some extent, this study’s results are applicable in clinical practice. The second limitation is the lack of information and control over medication use. The third limitation is the lack of a control group. However, we do not believe these two points would change this study’s conclusion because the immediate hypoalgesic effects of craniocervical flexion exercise have been consistently reported in previous studies [6–8] and the hypoalgesic effect of cervical retraction exercise has been considered comparable or greater than that of craniocervical flexion exercise.

## Conclusion

This study provided a preliminary evidence that the cervical retraction exercise has a comparable or greater immediate neck analgesic effect than the craniocervical flexion exercise in young individuals who have the non-acute neck derangement syndrome with a DP for cervical retraction or extension.

## Data Availability

The data that support the findings of this study are available from the corresponding author, [HT], upon reasonable request.

## Clinical trials registry

University hospital Medical Information Network (UMIN000044425)
